# The Association of Agent Orange Exposure with the progression of monoclonal gammopathy of undetermined significance to multiple myeloma: a population-based study of Vietnam War Era Veterans

**DOI:** 10.1186/s13045-023-01521-6

**Published:** 2024-01-08

**Authors:** Lawrence W. Liu, Mei Wang, Nikhil Grandhi, Mark A. Schroeder, Theodore Thomas, Kristin Vargo, Feng Gao, Kristen M. Sanfilippo, Su-Hsin Chang

**Affiliations:** 1 Research Service, St. Louis Veterans Affairs Medical Center, 501 N. Grand Blvd Suite 300, St. Louis, MO 63103 USA; 2 City of Hope National Comprehensive Cancer Center, 1500 E. Duarte Rd, Duarte, CA 91010 USA; 3 Department of Medicine, Washington University School of Medicine, 660 S. Euclid Avenue, Campus Box 8056, St. Louis, MO 63110 USA; 4 Division of Public Health Sciences, Department of Surgery, Washington University School of Medicine, 660 S. Euclid Avenue, Campus Box 8100, St. Louis, MO 63110 USA

## Abstract

Herbicide and pesticide exposure [e.g., agent orange (AO)] is associated with an increased risk of multiple myeloma (MM) due to the contaminant, 2,3,7,8-tetrachlorodibenzo-*p*-dioxin (TCDD). However, it is unclear whether TCDD/AO exposure (AO exposure hereafter) increases the risk of progression of monoclonal gammopathy of undetermined significance (MGUS) to MM. We sought to evaluate the association in a nationwide study of US Veterans. A natural language processing algorithm was used to confirm MGUS and progression to MM. We included Veterans who were diagnosed with MGUS from 10/1/1999 to 12/31/2021 and served during the Vietnam War Era from 1/9/1962 to 5/7/1975. AO exposure was stratified according to three TCDD exposure levels: high (1/9/1962–11/30/1965), medium (12/1/1965–12/31/1970), or low (1/1/1971–5/7/1975). The association between AO exposure and progression was analyzed using multivariable Fine-Gray subdistribution hazard model with death as a competing event. The analytic cohort included 10,847 Veterans with MGUS, of whom 26.3% had AO exposure and 7.4% progressed to MM over a median follow-up of 5.2 years. In multivariable analysis, high exposure was associated with an increased progression rate (multivariable-adjusted hazard ratio 1.48; 95% confidence interval 1.02–2.16), compared to Veterans with no exposure. This information is critical to inform progression risk in patients diagnosed with MGUS and prior AO exposure. It is also applicable to MGUS patients with occupational TCDD exposure from herbicides and pesticides.

**Supplementary Information:**

The online version contains supplementary material available at 10.1186/s13045-023-01521-6.

**To the editor,**

Agent Orange (AO) exposure is associated with an increased risk of both MGUS and MM [1, 2]. The term “Agent Orange” has been broadly used to refer to all the herbicidal agents (i.e., Agents Pink, Green, Purple, White, Orange, and Blue) used during the Vietnam War Era (1/9/1962–5/7/1975). AO and herbicides are commonly contaminated by a carcinogenic compound, 2, 3, 7, 8-tetrachlorodibenzo-*p*-dioxin (TCDD) [1]. Agents Pink, Green, and Purple had almost 16 times the amount of TCDD compared to AO (up to 66ppm versus 13ppm for AO) [3] and were largely used from January 1962 to November 1965, whereas Agent Orange was used from December 1965 to December 1970 [3, 4]. From January 1971 to April 1975, these herbicides were no longer used but this period was included in the “AO exposure” definition of the Veterans Health Administration (VHA) [3, 4], possibly due to residual exposure. This demonstrates that different time periods within the AO exposure period may have different levels of TCDD exposure.

Risk of both MGUS and MM increase with increasing serum concentrations of TCDD [1]. In addition, in a single-center cohort study (*n* = 211), AO exposure was associated with an 11-fold increased risk of progression of MGUS to MM [5]. Analyzing the risk of MGUS progression, based on TCDD exposure, could inform cancer prevention. This study aims to assess the association between AO exposure and progression of MGUS to MM in Vietnam War Era Veterans using the nationwide VHA data.

A recent population-based VHA study using the same database did not find an association between AO exposure and MGUS progression [6]. However, outcomes research is dependent on the accuracy of both the exposure and the outcome definitions. Our study (1) identified patients with MGUS using natural language processing (NLP)-assisted classification models to confirm MGUS and MM diagnosis [7], the latter of which was further confirmed by receipt of MM-specific treatments (Additional file 1: Table S1) (2) defined AO exposure levels based on historical documentation of herbicidal agents sprayed and their TCDD concentrations during the Vietnam War era [3, 4]. These have increased the accuracy of the outcome and the exposure measures, compared to the published studies which largely relied on administrative coding alone.

After applying inclusion and exclusion criteria (Additional file 1: Fig. S1), the final analytic cohort included 10,847 patients with MGUS, among whom 2851 (26.3%) had AO exposure (see Table 1). Progression was observed in 9.6% in the high-exposure group, 7.3% in the medium exposure group, 6.1% in the low exposure group, and 7.4% in the AO unexposed group (*P* < 0.0001; Table 1). In the multivariable analysis using Fine-Gray subdistribution hazard, time-to-competing-event model with death as a competing event (see Additional file 1 for details), compared to those with no AO exposure, the high-exposure group had a multivariable-adjusted hazard ratio (aHR) of 1.48 (95% Confidence Interval [CI] 1.02–2.16; Table 2). There were no statistically significant differences in progression rate noted in the low and medium exposure groups, compared to the no AO exposure group (Table 2).

**Table 1:** Baseline characteristics of the analytic cohort of MGUS patients stratified by AO exposure status

	Exposure status^†^				Total	*P*-value^‡^
	High	Medium	Low	Unexposed		
N	292	2314	245	7996	10,847	
%	2.7	21.3	2.3	73.7	100.0	
Gender (%)						< .0001
F	0.0	0.04	0.0	2.3	1.7	
M	100.0	99.96	100.0	97.7	98.3	
Race (%)						< .0001
Black	33.6	30.3	26.5	38.7	36.5	
White	66.4	69.7	73.5	61.3	63.5	
BMI at MGUS (%)						< .0001
Underweight	1.0	1.0	0.0	1.7	1.5	
Normal weight	19.5	16.9	19.6	21.4	20.4	
Overweight	34.9	36.4	31.0	33.7	34.3	
Obese	44.5	45.6	49.4	43.2	43.9	
M-spike (%)						0.088
< 1.5 g/dL	73.6	74.6	74.7	71.8	72.5	
> = 1.5 g/dL	4.1	5.1	4.9	6.3	6.0	
Missing	21.7	19.3	20.6	21.9	21.5	
Ig subtype (%)						0.844
A	14.7	13.7	12.2	14.0	13.9	
G	85.3	86.3	87.8	86.0	86.1	
Age at MGUS Diagnosis (year)						< .0001
Median	71.7	68.6	66.9	68.7	68.7	
IQR (Q1–Q3)	67.2–74.9	64.6–71.6	63.3–69.2	64.3–73.0	64.4–72.6	
Charlson Comorbidity score						0.004
Mean	2.4	1.8	2.0	2.0	2.0	
Std	3.0	2.6	2.9	2.8	2.8	
Ever served in Army during Vietnam Era (%)						< .0001
No	52.1	33.4	39.6	44.5	42.2	52.1
Yes	48.0	66.6	60.4	55.5	57.8	48.0
Ever served in Marine Corps during Vietnam Era (%)						< .0001
No	81.5	84.8	84.1	90.2	88.6	
Yes	18.5	15.2	15.9	9.9	11.4	
Ever served in Air Force during Vietnam Era (%)						< .0001
No	86.0	91.4	85.3	82.8	84.8	
Yes	14.0	8.6	14.7	17.3	15.3	
Ever served in Navy during Vietnam Era (%)						< .0001
No	79.8	90.0	88.6	83.0	84.5	
Yes	20.2	10.0	11.4	17.0	15.5	
Outcome (%)						< .0001
Progression	9.6	7.3	6.1	7.4	7.4	
Death without progression	41.1	31.2	35.5	41.9	39.4	
Censored	49.3	61.5	58.4	50.7	53.1	

^†^High exposure was during 1/9/1962–11/30/1965, medium exposure was during 12/1/1965–12/31/1970, and low exposure was during 1/1/1971–5/7/1975. The reasoning for this stratification was that the herbicidal agents used in the first time period had the highest levels of TCDD, followed by the second time period. The last time period was when the herbicidal agents were no longer used, but the toxicity may linger [3, 4]

^‡^Chi-square tests were conducted to compare percentages for categorical variables, t-tests were used to compare means for continuous variables, and Kruskal–Wallis test was used to compare medians

*Ig* immunoglobulin, *IQR* interquartile range, *MGUS* monoclonal gammopathy of undetermined significance, *BMI* body mass index, *M-spike* monoclonal spike

**Table 2:** Multivariable analysis of progression rate of MGUS to MM

Parameter		aHR	95% Confidence Interval	*P*-value
AO Exposure				
	None		(reference)	
	High	1.48	1.02–2.16	0.04
	Medium	1.05	0.89–1.25	0.55
	Low	0.83	0.50–1.40	0.49
Age at MGUS Diagnosis (year)		0.97	0.96–0.98	< 0.0001
Gender				
	Female		(reference)	
	Male	0.84	0.51–1.37	0.49
Race				
	White		(reference)	
	Black	1.23	1.07–1.42	0.004
BMI Category				
	Obese	1.29	1.06–1.57	0.012
	Overweight	1.24	1.01–1.51	0.041
	Normal weight		(reference)	
	Underweight	0.58	0.26–1.31	0.19
M-spike (g/dL)				
	≥ 1.5	4.37	3.62–5.26	< 0.0001
	< 1.5		(reference)	
MGUS Type				
	IgA	1.53	1.28–1.83	< 0.0001
	IgG		(reference)	
CCI		0.96	0.93–0.98	0.003

A multivariable time-to-competing event analysis with death as a competing event was utilized (see Supplementary section)

*aHR* multivariable-adjusted hazard ratio, *IG* immunoglobulin, *BMI* body mass index, *M-spike* monoclonal spike, *CCI *Charlson commorbidity index

This study has a few limitations. Although our study has better accuracy on MGUS diagnosis and the date of diagnosis, MGUS development may be earlier than clinically identified. Moreover, our study could not assess the mechanism of AO on progression to MM. We were also unable to assess dose-dependence of AO exposure on progression to MM since we did not have access to serum TCDD levels.

Our results suggest a dose-dependent relationship between higher levels of AO/TCDD exposure and progression of MGUS to MM, with a 48% increased risk in patients with high AO exposure compared to the group without AO exposure. This information is critical to inform progression risk in patients diagnosed with MGUS and prior AO exposure during the high-exposure period. Although AO is no longer used, TCDD is still found in many pesticides, herbicides, and other manufactured chemicals [3, 4], which may explain why studies have found farmers as risk factors for developing MGUS and MM [8–12]. This may expand the implications of our study to non-Veterans with MGUS and occupational TCDD exposure who may warrant more frequent follow-up.

### Supplementary Information

**Additional file 1.** Supplemental file for detailed methods and results.

## Data Availability

The data came from the Veteran's Health Administration and is not publicly available.
