# Supplementary material for: The Association of Agent Orange Exposure with the progression of monoclonal gammopathy of undetermined significance to multiple myeloma: a population-based study of Vietnam War Era Veterans
Source: J Hematol Oncol. 2024 Jan 8;17:3. doi: 10.1186/s13045-023-01521-6 (PMC10773019; doi:10.1186/s13045-023-01521-6)
Supplement: Supplementary file 1 — Additional file 1. Supplemental file for detailed methods and results. [file 13045_2023_1521_MOESM1_ESM.pdf]

Supplemental file for detailed methods and results

METHODS

Data, study population, and design

A retrospective, cohort study using data from the U.S. Veteran Health Administration (VHA) system was conducted. We identified patients diagnosed with MGUS from 10/1/1999-12/31/2021 via the International Classification of Diseases, $9^{th}/10^{th}$ version (ICD-9/10) codes of 273.1/47.2, then confirmed their diagnosis status and dates by an established natural language processing (NLP) algorithm. Patients were considered to have MGUS if they had M-protein detected on serum protein electrophoresis (SPEP) with immunofixation confirmation as well as a mention of diagnosis in their clinical notes. This step is crucial as our prior studies utilizing manual data abstraction demonstrated that, at best, only 80-84% of patients with ICD codes for MGUS were confirmed to have MGUS on manual chart review. Institutional Review Boards at both Washington University School of Medicine and Veteran Affairs Saint Louis Healthcare System approved the study.

Analytic MGUS cohort

We included Veterans with an NLP-confirmed MGUS diagnosis within 1999-2021 (**Supplemental Figure 1)**. The year of 1999 was chosen because this was when the Corporate Data Warehouse (CDW) database, a centralized, electronic storage of diagnostic data, laboratory data on serum protein electrophoresis (SPEP), immunofixation, and other test results. We excluded Veterans with 1) progression within 1 year after MGUS diagnosis date since patients with progression within 1 year may have had MM at MGUS diagnosis; 2) missing SPEP or immunofixation data; 3) MGUS types other than IgG and IgA because IgM MGUS does not typically progress to MM and light chain MGUS does not reliably show up on SPEP; 4)

missing race; 5) race other than black or white due to a small sample size; 6) missing BMI data; 7) documented AO exposure but never served in the Army, Airforce, Marines, or Navy during the Vietnam Era; 8) no AO exposure whose birth years were outside of the range of the birth years for the AO exposed group; and 9) service time outside of the Vietnam War Era (01/09/1962-5/7/1975), for which the first entry date and last separation date of the service episodes were used to identify their service time. The reason for the last two exclusion criteria was to ensure that the two groups with and without AO exposure are similar in age and timeframe of service to ensure that the two groups had comparable follow-up time.

Unique patient identifiers were used to collect data on type of MGUS, race, sex, M-protein levels, weight, height, and comorbidities at the time of MGUS diagnosis, as well as AO exposure (see below). The most frequently reported height as well as weight within 1 year before and closest to MGUS diagnosis was used for BMI calculation. BMI categories were underweight (BMI <18.5 kg/m^2), normal weight (BMI 18.5-24.9 kg/m^2), overweight (BMI 25-29.9 kg/m^2), or obese (BMI ≥30 kg/m^2).

AO exposure

Data on prior AO exposure for the MGUS cohort were obtained from the VHA CDW Patient 3.1 Domain which utilized the station and time period of service to determine exposure. The Military Service Episode Legacy section was used to identify the branch of service and location of stationing. Duration of exposure was defined as the length of stationing in an area designated as having AO exposure during the Vietnam War Era. Patients were designated as potentially having AO exposure if they met all of the following criteria: AO exposure documented in CDW, AO location was designated as Vietnam, and if they ever served in the army, air force, navy, or marine corps during the date range of 1/9/1962-5/7/1975.

The exposure group was further split into three groups (high, medium, low) depending on their majority time of stationing: 1/9/1962-11/30/1965 (high-exposure), 12/1/1965-12/31/1970 (medium-exposure), and 1/1/1971-5/7/1975 (low-exposure). The reasoning for this stratification was that the herbicidal agents used in the first time period had the highest levels of TCDD, followed by the second time period (Stellman et al. 2003; Walker et al. 2014). The last time period was when the herbicidal agents were no longer used and the toxicity may persist. For this reason, patients whose entire time of stationing fell in the last time period were categorized as no exposure. Patients whose time of stationing includes the first and/or the second time period but the majority time fell into the third time period were categorized into the low exposure group, as these Veterans may have had minimal TCDD exposure.

Outcome measures

The outcome was time from MGUS diagnosis to progression to MM. The progression status and dates were detected by identifying patients in the analytic cohort who had an ICD-10 code for MM, C90.0, and also received MM-specific treatment (see Supplemental Table 1). Progression was then further confirmed by a published NLP algorithm (Wang et al. 2023). These confirmation steps were included due to prior studies demonstrating that ICD codes for MM may be only 58% accurate in capturing true MM (La et al. 2023). Follow-up was determined by the time of MGUS diagnosis to MM diagnosis, death, or being censored at the time of study (2/7/2023).

Statistical analyses

Summary statistics of the demographic and clinical characteristics stratified by AO exposure status (high, medium, low, and none) were computed. To compare patients across these four groups, for categorical variables, we used chi-square tests to examine differences in proportions; for continuous variables, we used Student t tests to examine differences in means and Kruskal-Wallis tests to examine the differences

in medians. To compare the cumulative incidence of progression between the AO exposure groups, the Gray's test was performed with death as a competing event.

The association of the AO exposure with progression of MGUS to MM was estimated using a multivariable Fine-Gray subdistribution hazard model with death as the competing event, adjusting for known covariates associated with progression. These covariates included: age at MGUS diagnosis, gender, race (White [reference], Black), M-protein (>1.5, ≤1.5 g/dL [reference]), MGUS subtype (Ig A, and Ig G [reference]), BMI category (underweight, normal [reference], overweight, obese), and Charlson comorbidity index (CCI), all at time of MGUS diagnosis.

All tests were two-sided. Statistical significance was determined by an alpha level of 0.05. All statistical analyses were performed using SAS version 9.2 (SAS Institute Inc., Cary, NC).

RESULTS

After applying our NLP algorithm to confirm MGUS patients identified from ICD-9/10 codes from 1999-2021, we identified 42,979 patients (**Supplemental Figure 1)**. We excluded patients with progression within 1 year of MGUS diagnosis date (6,025), missing SPEP data (4,257), non-IgA/IgG MGUS (8,458, which includes IgM [5,371], Light Chain [2,269], IgE [6], IgD [8], or Biclonal [804] subtype), missing race (1,726), race other than Black or White (409), missing BMI data (54), no AO exposure and birth years outside of the birth year range of the AO exposed group (4,395), no service during the Vietnam Era (6,662), and documented AO exposure in CDW but never served in the Army, Airforce, Marines, or Navy during the Vietnam Era (146). This led to our final analytic cohort of 10,847 Veterans.

Of these patients, there were 292 (2.7%) patients in the high AO exposure group, 2,314 (21.3%) patients in the medium AO exposure group, 245 (2.3%) patients in the low AO exposure group, and 7,996 (73.7%) in the group without AO exposure (**Table 1)**.

The four groups were different in gender (P<0.0001), race (P<0.0001), BMI at MGUS diagnosis (P<0.0001), age at MGUS diagnosis (P<0.0001), Charlson Comorbidity Index (P=0.004), service branch (P<0.0001) and progression rate (P<0.0001). The median age of MGUS diagnosis was 71.7 years in the high exposure group, 68.6 years in the medium exposure group, 66.9 years in the low exposure group, and 68.7 years in the group without AO exposure (P<0.0001). The four groups were not statistically significantly different in proportion of patients with M-spike ≥1.5 g/dL (P=0.08), Ig subtype (P=0.84), or follow-up time (P=0.76).

Among the analytic MGUS cohort, 9.6% in the high exposure group, 7.3% in the medium exposure group, 6.1% in the low exposure group, and 7.4% in the AO unexposed group progressed (P<0.0001). However, no statistically significant difference in the cumulative incidence of progression was detected (p=0.54, **Supplemental Figure 2)**. However, in the multivariable analysis, the high exposure group had a significant increase in the progression rate from MGUS (multivariable-adjusted Hazard Ratio [aHR] 1.48; 95% Confidence Interval [CI] 1.02-2.16; **Table 2**) compared to those with no AO exposure. There were no significant differences in progression rate noted in the medium (aHR 1.05; 95% CI 0.89-1.25) and the low (aHR 0.83; 95% CI 0.50-1.40) exposure groups compared to no AO exposure.

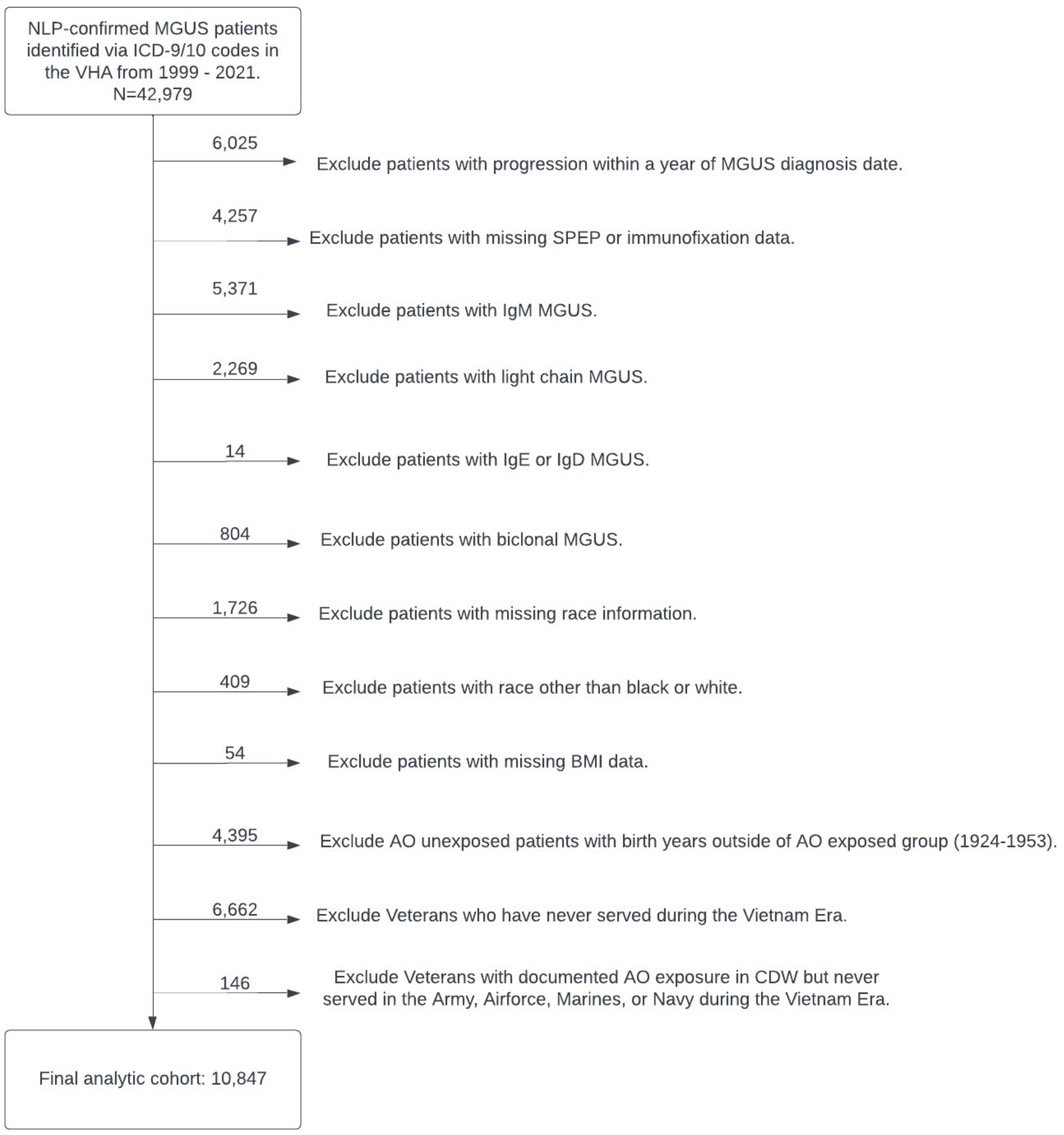

Supplemental Figure 1. Attrition Diagram.

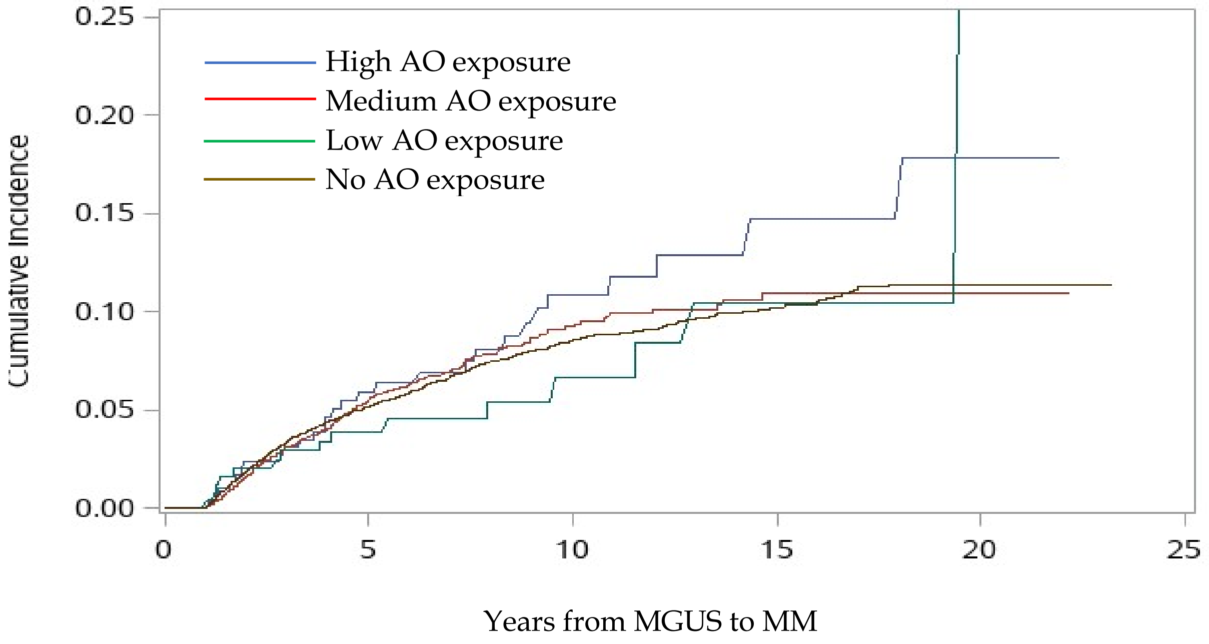

Supplemental Figure 2. Cumulative Incidence Function of Progression in patients with MGUS

Supplemental Table 1. List of MM-specific treatments:

abecma
belamaf
belantamab
belmaf
bendamustine
blenrep
bortezomib
carfilzomib
carvykti
cevostamab
ciltacabtagene
cilta-cel
cyclophosphamide
cytoxan
daratumumab
darzalex
elotuzumab
elranatamab
empliciti

evomela
farydak
idecabtagene
ide-cel
isatuximab
ixazomib
kyprolis
lenalidomide
melflufen
melphalan
ninlaro
panobinostat
pomalidomide
pomalyst
revlimid
sarclisa
selinexor
talquetamab
teclistamab
tecvayli
thalidomide
thalomid
velcade
venclexta
venetoclax
xpovio
